# Energy metabolism manipulates the fate and function of tumour myeloid-derived suppressor cells

**DOI:** 10.1038/s41416-019-0644-x

**Published:** 2019-12-10

**Authors:** Cong Hu, Bo Pang, Guangzhu Lin, Yu Zhen, Huanfa Yi

**Affiliations:** 1 Central Laboratory, The First Hospital of Jilin University, 130031, Changchun, Jilin China; 2 Key Laboratory of Organ Regeneration and Transplantation, Ministry of Education, 130021 Changchun, Jilin China; 3 Center for Reproductive Medicine, Center for Prenatal Diagnosis, The First Hospital of Jilin University, 130021, Changchun, Jilin China; 4 Department of Cardiology, The First Hospital of Jilin University, 130031, Changchun, Jilin China; 5 Department of Dermatology, The First Hospital of Jilin University, 130021, Changchun, Jilin China

**Keywords:** Immunoediting, Immunoediting

## Abstract

In recent years, a large number of studies have been carried out in the field of immune metabolism, highlighting the role of metabolic energy reprogramming in altering the function of immune cells. Myeloid-derived suppressor cells (MDSCs) are a heterogeneous population of cells generated during a large array of pathological conditions, such as cancer, inflammation, and infection, and show remarkable ability to suppress T-cell responses. These cells can also change their metabolic pathways in response to various pathogen-derived or inflammatory signals. In this review, we focus on the roles of glucose, fatty acid (FA), and amino acid (AA) metabolism in the differentiation and function of MDSCs in the tumour microenvironment, highlighting their potential as targets to inhibit tumour growth and enhance tumour immune surveillance by the host. We further highlight the remaining gaps in knowledge concerning the mechanisms determining the plasticity of MDSCs in different environments and their specific responses in the tumour environment. Therefore, this review should motivate further research in the field of metabolomics to identify the metabolic pathways driving the enhancement of MDSCs in order to effectively target their ability to promote tumour development and progression.

## Background

Cells use different energy metabolic pathways to produce ATP and to synthesise intermediates to support their growth, proliferation, function, and survival.^1^ In addition to the three main energy metabolic pathways that produce ATP – glycolysis, the tricarboxylic acid (TCA) cycle, and oxidative phosphorylation (OXPHOS) – there are three other pertinent pathways: the pentose-phosphate pathway (PPP), glutaminolysis, and fatty acid oxidation (FAO).^2^ Among the key energy metabolism pathways listed above, glycolysis and the PPP occur in the cytosol; the TCA cycle, OXPHOS, and FAO are limited to the mitochondria; and glutaminolysis progresses in both the cytosol and mitochondria.^3,4^ Cancer cells rely on glycolysis to produce ATP even under aerobic conditions, which is known as the Warburg effect. Aerobic glycolysis is a central requirement for meeting the bioenergetic and biosynthetic needs of rapidly proliferating cancer cells.^5–7^ This metabolic change of cancer cells may not only have an impact on immune cells but also the immune cells themselves can cause metabolic reprogramming to drive their activation and differentiation in response to pathogen-derived or inflammatory signals, thereby supporting distinct immune effector functions.^5,8^ Indeed, each subset of immune cells has distinct metabolic requirements at different stages of differentiation. Increased glycolysis has been associated with inflammatory-effector phenotypes in activated immune cells, whereas mitochondrial oxidation pathways such as FAO and OXPHOS are associated with quiescent, memory, and suppressive immune cells. Mounting evidence points to distinct mechanisms of energy metabolic regulation in immune cells according to the disease or stage of disease. Immune cells cannot only withstand the metabolic changes occurring in response to pathogen-derived signals, but these metabolic processes may themselves modulate the function and fate of these cells in a process referred to as energy metabolic reprogramming.^9,10^

Myeloid-derived suppressor cells (MDSCs) are a heterogeneous population of immature cells derived from myeloid progenitors with immunosuppressive functions.^11^ MDSCs have recently entered the research spotlight owing to an increasing recognition of their strong association with various pathological conditions, such as cancer, infectious diseases, trauma, autoimmune diseases, and transplantation. Under the influence of tumour-derived factors, myeloid cells are hijacked to become MDSCs that not only inhibit antitumour T-cell functions but also accelerate tumour progression by promoting angiogenesis, cell invasion, and the formation of pre-metastatic niches.^12^ Moreover, the ability of MDSCs to inhibit the antitumour function of T cells and natural killer (NK) cells in the context of tumour-associated inflammation is well established.^13^ Although the proportion of MDSCs is closely associated with clinical outcomes and therapeutic effects in patients with solid tumours,^14^ their roles in other diseases remain controversial. For example, MDSCs were shown to attenuate disease severity in murine models of autoimmune diseases through their T-cell suppression function, whereas other studies showed a deleterious role of MDSCs in disease progression.^15–17^ The specific factors contributing to these apparently different functions of MDSCs in different environments and contexts remain somewhat elusive. However, an ability for metabolic reprogramming and plasticity according to environmental requirements appears to play a role in this variation.

To date, the immune-metabolic pathways in T cells, macrophages, and dendritic cells (DCs) in immune responses have been widely reviewed;^18–21^ however, those of MDSCs are only recently coming to light, suggesting different energy metabolic pathways that affect MDSC differentiation and function. In this review, we discuss the evidence accumulated on energy metabolism in MDSCs and explore the potential relationships among differentiation, function, and immune metabolism and their influence on tumour development and progression.

## Phenotypes, differentiation, and biological functions of MDSCs

MDSCs rapidly expand during the disease course of cancer and the multiple chronic inflammatory conditions described above. MDSCs comprise two major subsets, including monocytic (M-MDSCs) and granulocytic (G-MDSCs) populations, which share an immature state and the ability to suppress adaptive immunity. In mice, MDSCs are characterised by the expression of CD11b and Gr1 and can be subdivided into M-MDSCs and G-MDSCs based on their expression of Ly6C and Ly6G as CD11b^+^Ly6C^high^Ly6G^−^ and CD11b^+^Ly6G^+^Ly6C^low^^22^ cells, respectively. Human MDSCs can be approximately classified as HLA-DR^low/−^CD33^+^, with M-MDSCs defined as CD11b^+^CD33^+^CD14^+^HLA-DR^−^ and G-MDSCs defined as CD11b^+^CD33^+^CD15^+^/CD66^+^HLA-DR^−^.^23,24^ Therefore, M-MDSCs and G-MDSCs may represent monocytes and granulocytes, respectively, with immunosuppressive properties. In addition, a recent study identified a novel subset of human MDSCs in metastatic paediatric sarcomas with a fibroblast phenotype, known as fibrocytic MDSCs, which are characterised by the expression of HLA-DR and their suppressive activity with indoleamine 2,3-dioxygenase (IDO); however, research on this subpopulation remains relatively scarce.^25,26^

Numerous factors are involved in the expansion and differentiation of MDSCs, although the molecular mechanisms underlying these activities are still widely debated. Tumours or the bone marrow stroma produce several mediators, including granulocyte/macrophage colony-stimulating factor (GM-CSF), granulocyte colony-stimulating factor (G-CSF), macrophage colony-stimulating factor (M-CSF), stem cell factor, vascular endothelial growth factor, and the alarmins S100A9 and S100A8, to induce or mobilise MDSCs through the major transcriptional factors/regulators signal transducer and activator of transcription 3 (STAT3), STAT5, CCAAT/enhancer-binding protein β, and NOTCH.^27,28^ This activation in turn induces inflammatory cytokines, including interferon-γ (IFN-γ), interleukin (IL)-1β, IL-4, IL-6, IL-13, and tumour necrosis factor-α (TNF-α), which endow MDSCs with their suppressive activity through interactions with nuclear factor κB (NF-κB), STAT1, and STAT6.^29–31^ Furthermore, retinoic acid-related orphan receptor 1, a member of the nuclear receptor superfamily, has been suggested to play an important role in the accumulation of MDSCs.^32,33^

MDSCs-mediated immunosuppressive mechanisms have been well defined and primarily include (1) nutritional deprivation, e.g. l-arginine (Arg) and l-cysteine (Cys) depletion and impaired T-cell viability, migration, and activation;^34^ (2) indirect suppression of T cells or effector B cells through inducing other tolerogenic immune cells such as regulatory T cells, regulatory B cells, and tumour-associated macrophages (TAMs);^35,36^ (3) production of inhibitory cytokines (e.g., TGF-β or IL-10) or the expression of inhibitory molecules to suppress T-cell responses;^37^ (4) synthesis of peroxynitrite or reactive oxygen species (ROS) to disturb IL-2 and T-cell receptor signalling;^38^ and (5) killing NK cells or inhibition of their function.^39^

## Energy metabolism in MDSCs

MDSCs are characterised by their myeloid origin, immature state, and immunosuppressive ability and show distinct functions and phenotypes in different diseases. Isolated tumour-derived MDSCs may appear to be functional in vitro, but this may not reflect their actual activity in the endogenous immune environment. MDSCs also display a certain degree of plasticity, assuming a classically activated or alternatively activated phenotype with pro-inflammatory or pro-tumour functions, respectively.^40,41^ Although the mechanisms of MDSC maturation and activation are well established, those determining the metabolic status and physiological metabolic reprogramming remain poorly understood. Available data suggest that the phenotypic heterogeneity of tumour-derived MDSCs is under the control of elements of energy metabolic pathways, including oxygen levels, FA metabolism, and inflammatory parameters, which may in turn regulate the balance between OXPHOS and glycolysis in MDSCs.^33,42^ Below, we provide an overview of each of the key metabolic pathways in MDSCs and their potential roles in mediating the tumour microenvironment and immune response.

### Glucose metabolism in MDSCs

The metabolic reprogramming of cancer cells, such as the use of aerobic glycolysis (the Warburg effect), affects the tumour microenvironment and infiltrating immune cells through changes in glucose metabolism.^43^ M-MDSCs can differentiate into M1- or M2-like TAMs and to TNF-α- and inducible nitric oxide synthase (iNOS)-producing DCs in the tumour environment, while monocytes also convert into M-MDSCs.^44–49^ During maturation and activation, these tumour-derived MDSCs exhibit an increase in central carbon metabolism, including glycolysis, the PPP, and the TCA cycle. G-MDSCs were also reported to utilise both glycolysis and OXPHO in a nasopharyngeal carcinoma tumour-bearing mouse model.^50^ Dynamic metabolic flux analysis, a unique tool that can quantify extracellular and intracellular nutrient and metabolite concentrations, demonstrated that MDSCs exhibit the Warburg effect during their maturation with high glucose and glutamine uptake rates, a reduced oxygen consumption rate (OCR) and that approximately 95% of the ATP generated was obtained through a glycolysis-dependent mechanism.^51^ Tumour-derived MDSCs exhibit high glycolysis upregulation, and its metabolite phosphoenolpyruvate could protect MDSCs from apoptosis and contribute to their survival.^38^ Owing to the high glucose uptake rates of both tumour cells and MDSCs, immune cells do not have any metabolic elasticity to acclimate to the condition of low oxygen tension and limited glucose availability, which could result in immune cell dysfunction and death, indirectly facilitating tumour escape and progression.

Various signalling pathways and transcription factors co-operate to control metabolic reprogramming in immune cells. The phosphatidylinositol 3-kinase (PI3K)–serine threonine protein kinase (AKT)–mammalian target of rapamycin (mTOR) pathway supports cellular proliferation through anabolism, with high rates of glycolysis and glutaminolysis for the synthesis of proteins and nucleic acids.^52,53^ Hypoxia-inducible factor 1α (HIF-1α), which is downstream of the PI3K–AKT–mTOR pathway, is activated in hypoxic environments.^54^ Under hypoxia, mTOR signalling activates the HIF-1α-mediated transcriptional programme, resulting in increased expression levels of glucose, lactate transporters, and glycolytic enzymes, accompanied by reduced mitochondrial oxygen consumption, subsequently mediating the switch from OXPHOS to glycolysis.^55,56^ As a crucial driver of glycolysis under hypoxic conditions, HIF-1α upregulation has also been shown to significantly enhance the suppressive function of MDSCs in tumours.^57^ Moreover, lactate, a product of glycolysis, increases the percentage of MDSCs and strengthens their suppressive activity via the HIF-1α pathway.^58^ In addition, inhibition of glycolysis by 2-deoxyglucose was shown to suppress the differentiation of MDSCs from precursor cells, whereas enhancement of glycolysis with metformin significantly rescued the rapamycin-induced decline of MDSCs.^59^ Furthermore, another study showed that deficiency in NAD-dependent deacetylase sirtuin 1, an enzyme that is responsible for deacetylating the proteins responsible for cellular regulation, could potentiate the glycolytic activity of MDSCs, contributing to the immunosuppressive function and pro-inflammatory ability of MDSCs in lymphoma and melanoma mouse models.^57^

In contrast to HIF-1α, AMP-activated protein kinase (AMPK) exerts immunosuppressive function by inhibiting glycolysis through the PI3K–AKT–mTOR pathway. AMPK drives glycolysis toward OXPHOS during glucose metabolism.^60,61^ Interestingly, AMPK is activated in MDSCs. Blocking the activity of AMPK in GM-CSF/IL-6-induced MDSCs resulted in reduced inhibitory function of the cells.^62^ In the tumour microenvironment, glycolysis activation induces bone marrow-derived myeloid precursor cells to expand to MDSCs, which could be inhibited by the mTOR inhibitor rapamycin.^63^

Besides glycolysis, glutaminolysis also ensures an adequate supply of intermediates and energy during tumour progression, and a recent study showed that glutaminolysis supports the maturation and immunosuppressive function of MDSCs through iNOS activity in vitro.^64^ Thus accumulating evidence now points to the high metabolic plasticity of immune cells, which can change their differentiation and function according to the context required.

### Lipid metabolism in MDSCs

Alterations of lipid metabolism pathways were recently suggested to be associated with increased haematopoietic activity and immunity.^65^ FAO, a process that produces acetyl-CoA that participates in the TCA cycle and OXPHOS, facilitates the generation of substantial amounts of ATP while providing the biosynthetic intermediates to support the generation of riboses, FA, and amino acid (AA). Recently, tumour-associated G-MDSCs and M-MDSCs were shown to undergo metabolic reprogramming by significantly increasing FAO as a primary energy source as opposed to glycolysis.^66^ This metabolic reprogramming is characterised by an increased mitochondrial mass and FA uptake, upregulated expression of the scavenger receptors FA translocase CD36 and recombinant macrophage scavenger receptor 1, enhanced levels of carnitine palmitoyltransferase 1 and 3-hydroxyacyl-CoA dehydrogenase, and an augmented OCR.^66^ This FAO upregulation is paralleled by the enhanced immunosuppressive function of MDSCs. FA transport protein (FATP), as a long-chain FA transporter, controls the suppressive activity of G-MDSCs through increased uptake of arachidonic acid and synthesis of prostaglandin E2, whereas deletion of the *FATP* gene abrogates their suppressive activity. Importantly, FAO inhibition was shown to restrain the immunoregulatory pathways along with the functions of tumour-infiltrating MDSCs, resulting in a T-cell-dependent delay in tumour growth, especially when combined with chemotherapy and adoptive cellular therapy.^66,67^ MDSCs are significantly increased in the peripheral blood and tumours in patients with multiple types of cancer, but only tumour-infiltrating MDSCs from colon adenocarcinoma and breast ductal carcinoma have been found to exhibit increased FA uptake and prefer using FAO to generate an adequate supply of ATP.^68–70^ The intracellular accumulation of lipids also activates the immunosuppressive function of MDSCs, which is inhibited after the genetic depletion of CD36.^71,72^ Furthermore, liver X receptor (LXR) serves as a critical regulator of lipid homoeostasis by driving the expression of key genes involved in cholesterol, FA, and glucose metabolism through NF-κB/IL-9 signalling.^73^ In ovarian cancer or melanoma, activation of the LXR/apolipoprotein E axis in MDSCs plays a role in inhibiting T cells both in vivo and in vitro.^74^

Taken together, MDSCs rely on FAO as the major metabolic fuel for the production of inhibitory cytokines. Consequently, targeting FAO may be a useful approach to limit the immune-suppressive function of MDSCs. However, the specific factors responsible for this shift among the TCA, glycolysis, and FAO pathways in the tumour microenvironment and the molecular networks involved in the energy metabolic reprogramming of MDSCs are still unknown.

### AA metabolism in MDSCs

Although the different immunosuppressive pathways of MDSCs in the cancer microenvironment may work simultaneously to exert their effects, increased AA metabolism appears to be a key requirement for immune tolerance. The metabolism of AAs, especially Arg, tryptophan (Trp), and Cys, plays an important role in the viability, migration, and activation of T cells.

Arg is a semi-essential AA that is only required by mammals under special circumstances such as for activating immune responses.^75^ In myeloid cells, Arg is actively metabolised through two pathways: the production of urea and l-ornithine (Orn) by arginase-1 (Arg-1) or the production of nitric oxide (NO) and l-citrulline by iNOS. Accordingly, a significant hallmark of MDSCs is the expression of both Arg-1 and iNOS to regulate Arg metabolism and to impair T-cell immune responses.^76,77^ In addition, Orn participates in the biosynthesis of polyamines and proline, which may play an important role in proliferating cells. Polyamines themselves can inhibit the expression of pro-inflammatory genes, thereby reducing iNOS protein expression.^78^ Polyamines also have the effects of promoting tumour growth through inhibiting T cells.^79^ In mouse models of neuroblastoma, blockage of the uptake or synthesis of polyamines markedly inhibited tumour progression, whereas elevation of the polyamine level significantly promoted tumour proliferation, infiltration, and invasion phenotypes.^80^ Although the function of T helper type 17 (Th17) cells in tumour progression remains controversial, there is now ample evidence to support their pro-tumour effect, and iNOS levels are correlated with Th17 induction in ovarian cancer patients.^81^

In different microenvironments, such as those with different pH values, the activities of Arg-1 and iNOS are distinctly different.^82^ This Arg paradox related to the activity of iNOS largely depends on the balance between extracellular and intracellular Arg concentrations.^83,84^ Quantitative proteomics and transcriptomic analyses have also been applied to explore the distinct gene expression profiles and substantial differences between G-MDSCs and neutrophils in tumour-bearing mice.^85,86^ Compared with naive neutrophils, both tumour-associated neutrophils and G-MDSCs showed downregulated expression of neutrophilic granule protein, and the differentially expressed genes were mostly enriched in pathways related to immune responses and processes as a whole, including inflammatory responses and cytokine activity. Pro-inflammatory cytokines (e.g. IFN-γ, TNFα, IL-1, and IL-12) secreted by M-MDSCs induce NO production and inhibit Arg-1 activity, whereas anti-inflammatory cytokines such as IL-4, IL-13, and IL-10 secreted by G-MDSCs increase Arg-1 activity and inhibit iNOS expression.^87,88^ Therefore, in general, G-MDSCs express higher levels of Arg-1, while M-MDSCs express higher levels of iNOS.^89,90^

Extracellular Arg can be transported into MDSCs by the cationic AA transporter 2B or is consumed via the secretion of Arg-1.^91,92^ Arg starvation impairs the function of T cells through both downregulating the expression of CD3ζ and inhibiting T cells in the G0–G1 phase of the cell cycle.^93,94^ NOHA and nor-NOHA, two Arg-1 inhibitors, reduce this suppressive function of MDSCs and enhance T-cell-mediated antitumour immunity.^95^ The induction of iNOS in M-MDSCs and endothelial nitric oxide synthase (eNOS) in G-MDSCs also plays a key regulatory role during Arg metabolism^96^ and impairs T-cell responses through independent NO-related pathways. NO has a direct pro-apoptotic effect on T cells by impairing the STAT5 and Akt IL-2 receptor signalling pathways.^97^ In G-MDSCs, NO can react with ROS such as superoxide and hydrogen peroxide to produce peroxynitrite, which can induce the apoptosis of T cells and prevent their recruitment.^98^ Overall, these findings demonstrate that Arg metabolism by Arg-1, iNOS, and eNOS represents a major regulatory pathway in MDSCs; thus manipulation of these regulators may become a new therapeutic approach to treat tumours.^99^ However, more details of the regulation mechanism in the tumour environment remain to be elucidated. For example, the functions of G-MDSCs and M-MDSCs in different microenvironments are still unclear, and competition between Arg-1 and iNOS for Arg may be relevant. In addition, the balance between Arg-1 and iNOS expression may affect pro-inflammatory or anti-inflammatory responses in MDSCs.

Another relevant AA pathway in MDSCs involves the metabolism of Trp, an essential AA in mammals. Trp is a substrate of indoleamine 2,3-dioxygenase 1 (IDO1) that catalyses the first rate-limiting step in the kynurenine (Kyn) pathway, resulting in Trp depletion and the production of a series of immunoregulatory molecules collectively known as Kyn and serotoninc.^100^ Early studies indicated that IFN-γ induced IDO1 in tumour cells, which consumed Trp, in turn inhibiting T-cell proliferation.^101^ MDSCs also decrease the levels of Trp via the expression of IDO1 in the external environment to impair cytotoxic T-cell responses and survival.^100,102,103^ Trp starvation arrests T-cell growth via the general control non-repressed 2 pathway. Moreover, Kyn production has an immunoregulatory effect via activating aryl hydrocarbon receptor (AhR), whereas some human microbiomes can also activate AhR to induce IDO1 expression and the anti-inflammatory response.^104^ Moreover, a recent study showed that IDO played a role in MDSCs-driven immune escape and autochthonous carcinoma progression.^100^

**Fig. 1:**
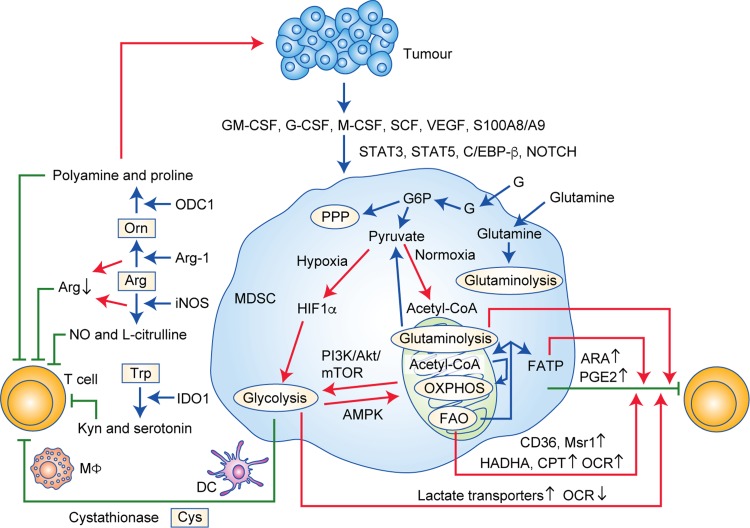
Energy metabolism contributes to the immunosuppressive role of MDSCs. In the tumour microenvironment, GM-CSF, G-CSF, M-CSF, SCF, VEGF, and S100A8/A9 mobilise MDSCs through the pathways of the major transcriptional factors/regulators STAT3, STAT5, C/EBP-β, and NOTCH. MDSCs uptake glucose to the cytosol, which is then processed to pyruvate. Under normoxia, the pyruvate is converted into acetyl-CoA, as the fuel of OXPHOS and a crucial producer of FAO in the mitochondria. Under hypoxia, HIF-1α as a catabolite facilitates glycolysis, and the AMPK pathway drives glycolysis towards OXPHOS, while the PI3K–Akt–mTOR pathway promotes glycolysis. The downstream production of glucose, lipids, and AAs drives the impressive activities of MDSCs in different ways. GM-CSF granulocyte-macrophage colony-stimulating factor, G-CSF granulocyte colony-stimulating factor, M-CSF macrophage colony-stimulating factor, SCF stem cell factor, VEGF vascular endothelial growth factor, C/EBP-β CCAAT/enhancer-binding protein β, G glucose, G6P glucose-6-phosphate, PPP pentose-phosphate pathway, OXPHOS oxidative phosphorylation, FAO fatty acid oxidation, HIF-1α hypoxia-inducible factor 1α, FATP fatty acid transport protein, Msr1 recombinant macrophage scavenger receptor 1, CPT1 carnitine palmitoyltransferase 1, HADHA 3-hydroxyacyl-CoA dehydrogenase, OCR oxygen consumption rate, ARA arachidonic acid, PGE2 prostaglandin E2, Arg arginine, Orn ornithine, Trp tryptophan, Cys cysteine, ODC1 ornithine decarboxylase1, Arg-1 arginase-1, iNOS inducible nitric oxide synthase, IDO1 indoleamine 2,3-dioxygenase 1, Mɸ macrophages, DC dendritic cells

Cys is another essential AA required by mammalian cells for protein synthesis and proliferation, which is generated through two main pathways. In the first pathway, cystine (Cys-Cys) is transported from the extracellular environment into cells that express a plasma membrane Cys-Cys transporter and is then reduced to Cys in the intracellular environment. In the other pathway, cells convert intracellular methionine into Cys through cystathionase activity.^105^ Cys was also found to be required for T cells during antigen presentation and activation.^106^ Since T cells lack cystathionase and do not have a transporter to import Cys-Cys or Cys from the extracellular environment, they only obtain Cys that is exported from cells such as macrophages and DCs.^107^ MDSCs can take up Cys from their extracellular environment but do not export Cys-Cys, which is also sequestered from macrophages and DCs. Therefore, DCs and macrophages cannot provide Cys to T cells in the presence of MDSCs, resulting in the lack of tumour-specific T-cell activity to suppress antitumour immunity,^108^ further highlighting the potential of MDSCs as a therapeutic target (Fig. 1).

## Perspectives and conclusions

Accumulating evidence suggests that the regulation of cellular metabolism essentially dictates the phenotype and function of immune cells. In most cases, such metabolic reprogramming is triggered by a key receptor signalling event along with the metabolite availability status and adapts according to the microenvironment. The metabolic reprogramming of MDSCs enhances their population while promoting tumour growth and cancer progression. In the tumour environment, M-MDSCs prefer FAO over glycolysis to supply ATP, whereas G-MDSCs preferentially utilise glycolysis and OXPHO. In AA metabolism, G-MDSCs express higher levels of Arg-1, while M-MDSCs express higher levels of iNOS to catabolise Arg to exert the inhibition function of MDSCs. However, elucidating the details of MDSC metabolism in the tumour microenvironment requires further exploration, including determining the difference between G-MDSCs and M-MDSCs, between M-MDSCs and macrophages, and between G-MDSCs and neutrophils. Moreover, the differences in the activities and metabolic pathways of MDSCs in tumours and other diseases should be clarified. MDSCs represent a potential therapeutic target for cancer owing to their ability to suppress immune responses as well as their high plasticity and differentiation potential. The metabolism-related pathways in MDSCs that mediate immune suppression reviewed herein may be of considerable clinical importance for tumour treatment as a new therapeutic target. Accordingly, gaining a better understanding of the energy metabolism of MDSCs in the tumour environment may allow for more precise and targeted therapy, including blockade of MDSC migration to the tumours or inhibition of MDSC differentiation in tumour-associated cells.

## Data Availability

Not applicable.
